# Effects of Dietary Chitin Inclusion on Growth Performance, Intestinal and Hepatic Health, and Muscle Texture in Hybrid Grouper (*Epinephelus fuscoguttatus* ♀ × *E. lanceolatus* ♂)

**DOI:** 10.3390/biology15211954

**Published:** 2026-10-30

**Authors:** Wanjia Chen, Luyang Zhou, Rongpei Zhong, Feng Tang, Wenye Zhu, Yinzhao Zhang, Beiping Tan, Junming Deng

**Affiliations:** 1 College of Fisheries, Guangdong Ocean University, Zhanjiang 524088, China; bxg158595@163.com (W.C.); linxiangqin2025@163.com (R.Z.); zhuwenye@126.com (W.Z.); bptan@126.com (B.T.); 2 Zhejiang Yinghaoge Biological Co., Ltd., Zhoushan 316100, China; likeeee2020@163.com (L.Z.); jlz18276758998@163.com (F.T.); wc9264@163.com (Y.Z.)

**Keywords:** chitin, Antarctic krill meal, hybrid grouper, intestinal health, hepatic health

## Abstract

Antarctic krill (*Euphausia superba*) meal is a valuable aquafeed ingredient, but its chitin-rich exoskeleton may limit nutrient utilization. A 12-week trial evaluated fishmeal-based diets containing 0, 1.5%, 3.0%, or 6.0% chitin in hybrid grouper (*Epinephelus fuscoguttatus* ♀ × *E. lanceolatus* ♂). Dietary chitin did not significantly affect survival or growth. However, feeding rate and feed conversion ratio initially increased and then decreased, while protein efficiency ratio declined at all supplemented levels. At 1.5%, intestinal chitinase activity and IL-1β, TNF-α, and IL-6 were reduced. At ≥3.0%, serum T-BIL content, AST and ALT activities, AST/ALT ratio, plasma LPS content and DAO activity increased, whereas intestinal MPS, LZM, IgM, TGF-β1, IFN-γ, and IL-8 decreased. At 6.0%, lipase activity was further reduced. Some hepatic immune markers declined at 1.5% dietary chitin, while others decreased only at higher levels. In contrast, IL-10 increased at ≥3.0%, indicating altered hepatic immune homeostasis rather than generalized immunosuppression. Hepatic antioxidant capacity decreased at 6.0%. Dietary chitin ≥ 1.5% also reduced muscle hardness, gumminess, and chewiness. These findings indicate that even at the lowest tested inclusion level (1.5%), dietary chitin affected feed utilization and muscle texture, highlighting the need for further investigation of inclusion levels below 1.5%.

## Simple Summary

Aquaculture needs sustainable feed ingredients to reduce dependence on traditional fishmeal. Antarctic krill meal is a valuable feed ingredient, but it contains chitin, which may affect nutrient use by fish. This study investigated how different levels of dietary chitin influence the growth, health, and flesh quality of hybrid grouper. The results showed that chitin did not significantly affect fish survival or growth. However, higher dietary chitin inclusion levels were associated with changes in intestinal and hepatic health indicators, immune-related responses, and muscle texture. The observed changes in immune markers suggest altered immune regulation. Even at 1.5%, dietary chitin affected feed utilization and muscle texture, while higher inclusion levels were associated with additional intestinal and hepatic alterations. These findings suggest that controlling chitin levels is important when using krill-based ingredients in aquaculture feeds. The study provides useful information for developing safer and more effective diets for sustainable hybrid grouper farming.

## 1. Introduction

The rapid expansion of global aquaculture has resulted in a growing need for nutritionally valuable protein sources in aquafeeds. Diets for carnivorous aquaculture species still rely heavily on fishmeal because of its high protein digestibility and well-balanced amino acid profile, which closely matches the nutritional requirements of fish [1,2]. However, fishmeal supply and cost remain vulnerable to variability in fishery resources, climatic conditions, international trade, and market dynamics [3]. Therefore, identifying nutritionally adequate, functionally beneficial, safe, and consistently available alternative protein sources is essential for the sustainable development of aquaculture [4]. Antarctic krill (*Euphausia superba*) is an abundant marine resource occupying a low trophic level in the Southern Ocean [5]. Krill meal is rich in high-quality protein, phospholipids, astaxanthin, and other bioactive nutrients, conferring considerable nutritional value and potential for use in aquafeeds [6]. However, krill meal also contains relatively high levels of chitin [7,8]. Preliminary analyses conducted by our research group showed that the chitin content of Antarctic krill meal ranged from 7.90% to 8.43% (unpublished data). Chitin may limit the inclusion of krill meal and other crustacean-derived ingredients at high dietary levels in aquafeeds [9,10,11]. Chitin is an unbranched polysaccharide consisting of N-acetyl-D-glucosamine residues joined through β-(1 → 4) glycosidic linkages. Chitin occurs naturally as a major structural component of crustacean exoskeletons, insect cuticles, and fungal cell walls [12,13,14]. The nutritional effects of chitin in aquatic animals vary depending on the dietary dose and species. As chitin-containing ingredients, including insect meal, shrimp and crab shell meal, and krill meal, are increasingly incorporated into aquafeeds, their effects on fish growth, digestive function, intestinal microbiota, and immune responses have attracted increasing attention [15,16,17].

Dietary inclusion of chitin and its derivatives, including chitosan and chitooligosaccharides, at appropriate levels (0.5–2.0%) has been shown to promote growth performance and enhance immune responses in farmed fish [18,19]. However, because of its highly stable structure and poor water solubility, excessive dietary chitin may resist complete degradation in the digestive tract. These properties may impair nutrient digestion and absorption, reduce nutrient utilization efficiency, and disrupt intestinal function and metabolic homeostasis [9,15,20]. The capacity for chitin degradation and utilization varies markedly among fish species. For example, cobia (*Rachycentron canadum*) can efficiently utilize crustacean-derived chitin owing to its relatively high endogenous chitinolytic activity [21]. In contrast, dietary supplementation with exogenous chitinase was reported to enhance growth performance and immune competence in orange-spotted grouper (*Epinephelus coioides*) [22]. The available evidence suggests that the nutritional response of chitin depends not only on its dietary inclusion level but also on species-specific feeding habits, digestive physiology, and endogenous chitinolytic capacity. Previous ingredient-replacement studies have primarily focused on whole insect- or crustacean-derived ingredients. Because the use of these ingredients simultaneously alters dietary levels of proteins, lipids, minerals, and bioactive compounds, such studies cannot clearly distinguish the specific effects attributable to chitin [10,23]. Therefore, evaluating the effects of chitin independently under nutritionally controlled conditions is necessary to clarify whether chitin represents a major constraint on the high-level inclusion of krill meal in aquafeeds.

Hybrid grouper (*Epinephelus fuscoguttatus* ♀ × *E. lanceolatus* ♂) is a commercially important aquaculture species in Asia, valued for its rapid growth, superior flesh quality, high market value, and broad environmental tolerance. This study aimed to evaluate the effects of graded dietary chitin inclusion on growth performance, feed utilization, intestinal physiological responses, intestinal microbiota, hepatic health, and muscle texture in hybrid grouper, and to assess the practical relevance of the tested chitin levels to Antarctic krill meal utilization.

This study aimed to systematically evaluate the effects of graded dietary chitin inclusion on growth performance, feed utilization, intestinal digestive function and barrier integrity, intestinal microbiota, hepatic health, and muscle textural properties in hybrid grouper. The findings are expected to provide insights into the physiological responses to crustacean-derived chitin and inform future studies on Antarctic krill meal utilization.

## 2. Materials and Methods

### 2.1. Experimental Diets

A basal diet for hybrid grouper was formulated using fishmeal, cottonseed protein concentrate, and soybean meal as the primary protein sources. The basal diet was supplemented with 0%, 1.5%, 3.0%, or 6.0% chitin (product No. C104157, batch No. B2302336; Shanghai Aladdin Biochemical Technology Co., Ltd., Shanghai, China) to produce four isonitrogenous experimental diets with comparable gross energy contents (FM, C1.5, C3, and C6; Table 1). According to the manufacturer’s certificate, the chitin was derived from shrimp and crab shells (ash: 1.91%; molecular weight: 221.21 g/mol). The experimental diets were prepared and stored following the method described by Lin et al. [24].

### 2.2. Fish and Feeding Management

Juvenile hybrid grouper used in this study were obtained from a commercial farm near Dongnan Wharf, Zhanjiang, China, and acclimated to the control diet for 2 weeks. After 24 h of fasting, 360 healthy juveniles of uniform size (approximately 15.00 g) were randomly assigned to 12 fiberglass tanks (0.5 m^3^; 30 fish/tank), forming four dietary treatment groups with three replicate tanks each. Fish were fed to apparent satiation twice daily (08:00 and 17:00) for 12 weeks. Feed intake was recorded daily for each tank, and feces were removed daily. Throughout the trial, all tanks were maintained under the natural photoperiod in Zhanjiang, China, with an estimated light cycle ranging from approximately 11.7L:12.3D to 13.3L:10.7D. Water quality was maintained within the following ranges: temperature, 26–30 °C; dissolved oxygen, >6 mg/L; pH, 7.4–7.7; salinity, 26–30‰; and total ammonia nitrogen, <0.2 mg/L.

### 2.3. Sample Collection

At the end of the feeding trial, fish were deprived of feed for 24 h and anesthetized using eugenol diluted 1:10,000. Fish from each tank were subsequently enumerated and weighed to determine growth performance indices. Three fish per tank were sampled for whole-body composition analysis and stored at −20 °C, while another three were measured for body weight, body length, and visceral, intestinal, and liver weights to calculate morphometric indices. Blood was collected from two groups of four fish per tank via the caudal vein using 1-mL syringes. For serum preparation, blood was held overnight at 4 °C before centrifugation (4000 rpm, 10 min, 4 °C). For plasma preparation, blood was collected using sodium heparin as an anticoagulant (Shanghai Aladdin Biochemical Technology Co., Ltd., Shanghai, China) and centrifuged under the same conditions. Serum and plasma were stored at −80 °C for subsequent biochemical analyses.

After blood collection, six of the eight blood-sampled fish per tank were used for tissue biochemical, gene expression, and muscle texture analysis. Foregut samples from three fish were frozen in liquid nitrogen and stored at −80 °C until digestive enzyme activity assays. Hindgut and liver samples from all six fish were each divided into two portions: one frozen in liquid nitrogen and stored at −80 °C for enzyme assays, and the other preserved in RNAlater at −80 °C for mRNA expression analysis. Liver samples from the remaining two blood-sampled fish per tank were excised and fixed in 2.5% glutaraldehyde or 4% formaldehyde for ultrastructural and histological analyses, respectively. Hindgut tissue and contents from three separately sampled fish per tank in the FM and C6 groups were placed in sterile cryovials, snap-frozen in liquid nitrogen, and stored at −80 °C until intestinal microbiota analysis. Dorsal muscle samples from the same six fish used for biochemical and gene expression analysis were cut into approximately 2 × 1 × 1 cm pieces. These fresh muscle samples were stored at 4 °C without freezing and subjected to texture profile analysis at room temperature immediately after removal from refrigerated storage, with all measurements completed on the day of sampling. Other tissues were maintained under frozen conditions when required by the corresponding analytical procedures.

### 2.4. Sample Analysis

#### 2.4.1. Proximate Composition Determination

The proximate composition of whole-body fish and experimental diets was determined following AOAC methods [25]. Dry matter was measured by oven-drying at 105 °C to constant weight, crude protein by the Kjeldahl method using an automated nitrogen analyzer (K9860; Hanon, Jinan, China), crude lipid by Soxhlet extraction with petroleum ether at 60 °C, and crude ash by pre-carbonization followed by incineration at 550 °C for 6 h. Gross energy was determined with an oxygen bomb calorimeter (C 6000; IKA Werke GmbH & Co. KG, Staufen im Breisgau, Germany) after pelletizing the samples. Chitin content was determined by HPLC (1260 Infinity II; Agilent Technologies, Santa Clara, CA, USA) quantification of glucosamine released following hydrolysis with 6 mol/L HCl [26].

#### 2.4.2. Enzyme Activity and Biochemical Assays

Approximately 0.2 g of foregut, hindgut, or liver tissue was homogenized on ice in 0.9% physiological saline (1:9, *w*/*v*) and centrifuged at 3500 rpm for 10 min at 4 °C. Supernatants were collected, and total protein (TP) was measured using a commercial kit (A045-4-2; Nanjing Jiancheng Bioengineering Institute, Nanjing, China). Protein concentrations were normalized with physiological saline, and other biochemical parameters were measured using commercial kits according to the manufacturers’ instructions.

Kits from Nanjing Jiancheng Bioengineering Institute (Nanjing, China) were used to determine the serum total cholesterol (T-CHO; A111-1-1), triglyceride (TG; A110-1-1), total bilirubin (T-BIL; C09-1-1), and albumin (ALB; A028-2-1) levels, as well as aspartate aminotransferase (AST; C010-2-1), alanine aminotransferase (ALT; C009-2-1), and alkaline phosphatase (AKP; A059-2-1) activities.

Kits from Beijing Boxbio Science & Technology (Beijing, China) were used to determine foregut trypsin (AKPR005M), lipase (AKFA006C) and Na^+^/K^+^-ATPase (AKOP001M) activities. Hepatic malondialdehyde (MDA; AKFA013M) and glutathione (GSH; AKPR008M) levels, total antioxidant capacity (T-AOC; AKAO012M), superoxide dismutase (SOD; AKAO001C), and catalase (CAT; AKAO003-1M) activities were also determined.

ELISA kits from Shanghai Enzyme-linked Biotechnology Co., Ltd. (Shanghai, China) were used to determine plasma endothelin-1 (ET-1; ml027981) and lipopolysaccharide (LPS; ml662413) levels, and diamine oxidase (DAO; ml027888) activity; lysozyme (LZM; ml300815) activity and immunoglobulin M (IgM; ml300551) level in the serum, hindgut, and liver; transforming growth factor-β1 (TGF-β1; ml028281), interleukin-10 (IL-10; ml824689), interleukin-6 (IL-6; ml063159), interleukin-1β (IL-1β; ml106733), tumor necrosis factor-α (TNF-α; ml002095), interleukin-8 (IL-8; ml027347), and interferon-γ (IFN-γ; ml077386) levels in the hindgut and liver; and serum complement 3 (C3; ml003460) and complement 4 (C4; ml003461) levels. Hindgut mucin 2 (MUC2; ml107294), mucopolysaccharide (MPS; ml038490), and polymeric immunoglobulin receptor (pIgR; ml057740) levels and chitinase (ml076758) activity were determined, whereas hepatic reactive oxygen species (ROS; ml092661) levels were measured. For liver, foregut, and hindgut tissues, enzyme activities and analyte concentrations were normalized to tissue protein content and expressed per gram or milligram of protein, as appropriate.

#### 2.4.3. Histological Observation

Liver tissues were fixed in 4% formaldehyde for 24 h, transferred to 70% ethanol, dehydrated through graded ethanol, cleared with xylene, and embedded in paraffin. Tissue sections 5–7 μm thick were prepared, stained with hematoxylin and eosin (H&E), and mounted with neutral balsam. The sections were examined using a Nikon Eclipse Ni-U microscope (Nikon Corporation, Tokyo, Japan).

Liver samples (approximately 1 mm^3^) were fixed in 2.5% glutaraldehyde at 4 °C for 2–4 h, followed by post-fixation in 1% osmium tetroxide for 1–2 h. Samples were rinsed with phosphate buffer, dehydrated through graded ethanol, and embedded and polymerized in epoxy resin. Ultrathin sections (50–70 nm) were stained with uranyl acetate and lead citrate and examined by transmission electron microscopy (HT7700; Hitachi High-Tech Corporation, Tokyo, Japan) to assess hepatocyte ultrastructure.

#### 2.4.4. Gene Expression Analysis

Total RNA was isolated from hindgut and liver tissues using the TransZol Up Plus RNA Kit (TransGen Biotech Co., Ltd., Beijing, China). RNA integrity was verified by 1% agarose gel electrophoresis, whereas RNA purity and concentration were determined using a NanoDrop 2000 spectrophotometer (Thermo Scientific, Waltham, MA, USA). Subsequently, RNA was reverse-transcribed into cDNA using the PrimeScript™ RT Reagent Kit (Takara, Tokyo, Japan) according to the manufacturer’s protocol. Gene-specific primers were designed using Primer Premier 5.0 based on sequences retrieved from GenBank, and *β-actin* was used as the reference gene (Table 2). RT-qPCR was performed using the SYBR^®^ Premix Ex Taq™ II Kit (Takara, Tokyo, Japan) according to the manufacturer’s instructions. Relative target-gene expression was calculated using the 2^−ΔΔCt^ method [27].

#### 2.4.5. Intestinal Microbiota Analysis

Total DNA was extracted from hindgut samples, with spike-in DNA added for absolute quantification. PCR amplicons were purified, quantified, normalized, quality-checked, and sequenced on a NovaSeq 6000 sequencing platform (Illumina, Inc., San Diego, CA, USA). Raw reads were quality-filtered with Trimmomatic v0.33, primer sequences were trimmed with Cutadapt v1.9.1, and paired-end reads were merged and length-filtered using USEARCH v10. Internal-standard sequences were identified and removed by alignment using BLASTN v2.9.0+, after which denoising and chimera removal were performed using the DADA2 (version 1.18.2) algorithm implemented in QIIME 2 (version 2020.6) to generate high-quality nonchimeric sequences. A standard curve was generated from the internal-standard sequences to enable absolute quantification of microbial sequence copy numbers. Amplicon sequence variants (ASVs) were inferred using DADA2 and filtered according to predefined quality-control criteria. Taxonomic assignment was performed against the SILVA 138 database using BLAST alignment and Bayesian classification (minimum sequence identity: 90%; minimum coverage: 90%; consensus threshold: 51%; maximum accepted matches: 3; Bayesian confidence threshold: 0.7). Taxonomic composition was summarized at the phylum and genus levels, while principal coordinate analysis (PCoA) was used to visualize microbial community patterns. Observed ASV counts and taxonomic absolute-abundance profiles were examined descriptively. ASV counts were not standardized by rarefaction to a common sequencing depth. Thus, the reported ASV counts were based on the original observed ASV data.

#### 2.4.6. Muscle Texture

Muscle texture was determined using a texture analyzer ((TA.XTplusC; Stable Micro Systems Ltd., Godalming, UK) equipped with a cylindrical flat-ended probe (P/36; 36 mm in diameter). Dorsal muscle samples were collected from six fish per tank and cut into pieces measuring approximately 2 × 1 × 1 cm. After collection, fresh muscle samples were stored at 4 °C without freezing. Texture analysis was performed at room temperature immediately after the samples were removed from refrigerated storage, and all measurements were completed on the day of sampling. Each sample was positioned on the testing platform with the compression direction perpendicular to the muscle fiber orientation. Texture profile analysis (TPA) was performed using two consecutive compression cycles with a compression strain of 40% and a 5-s interval between cycles. The pre-test, test, and post-test speeds were all set at 60 mm/min, with a trigger force of 5 g. Textural parameters, including hardness, springiness, cohesiveness, adhesiveness, gumminess, chewiness, and resilience, were calculated from the force–time curves [28].

### 2.5. Statistical Analysis

All data were assessed for normality and homogeneity of variance before being subjected to one-way ANOVA using IBM SPSS Statistics 27.0 (IBM Corp., Armonk, NY, USA), followed by Duncan’s multiple range test for comparisons among treatment groups. The tank was considered the independent experimental unit, with three replicate tanks per treatment (*n* = 3). For tissue-related analyses, samples from multiple fish within the same tank were pooled before measurement. For parameters measured individually, tank-level means were calculated prior to statistical analysis. Linear and quadratic dose–response trends were analyzed based on dietary chitin inclusion levels. To reduce the risk of false-positive findings, *p*-values for treatment effects on cytokine and immune-related parameters were further adjusted using the Benjamini–Hochberg false discovery rate (FDR) procedure. Results are expressed as means ± standard errors of the mean (SEM). Statistical significance was defined as *p* < 0.05 or an FDR-adjusted *q*-value < 0.05, as appropriate for the statistical analysis performed.

## 3. Results

### 3.1. Growth Performance

SR ranged from 82.22% to 90.00% and did not differ significantly among dietary treatments (*p* > 0.05). Dietary chitin inclusion had no significant influence on growth performance or morphometric indices. As dietary chitin increased, FR and FCR initially increased and then declined, both peaking in the C1.5 group (*p* < 0.05). PER was significantly reduced at all chitin inclusion levels, with the minimum recorded in the C1.5 group. In addition, FR, FCR, and PER exhibited significant quadratic trends across dietary chitin levels (*p* < 0.05), whereas their linear trends were not significant (Table 3).

### 3.2. Whole-Body Composition

No significant differences were observed in whole-body crude protein, crude lipid, ash, or moisture contents among treatments (*p* > 0.05). Although none of these indices showed a significant overall ANOVA effect, ash content exhibited a significant positive linear trend (*p* < 0.05), whereas crude lipid showed a significant quadratic trend (Table 4).

### 3.3. Digestive Enzyme Activity

No significant differences were observed in foregut trypsin or Na^+^/K^+^-ATPase activities among treatments (*p* > 0.05). Hindgut chitinase activity was significantly reduced at dietary chitin levels of ≥1.5%, whereas foregut lipase activity was significantly decreased only at 6.0% dietary chitin (*p* < 0.05). Lipase and chitinase activities showed significant negative linear trends. Na^+^/K^+^-ATPase activity also exhibited a significant positive linear trend, whereas no significant quadratic trends were detected for any of the measured enzymes (Table 5).

### 3.4. Intestinal Mucosal Barrier Function

#### 3.4.1. Intestinal Mucosal Permeability

Plasma LPS content and diamine oxidase (DAO) activity progressively increased with increasing dietary chitin level (*p* < 0.05), whereas ET-1 content tended to increase but did not differ significantly among treatments. Plasma LPS content and DAO activity in the C1.5 group did not differ significantly from those in the FM group. LPS content and DAO activity also exhibited significant positive linear trends across dietary chitin levels, whereas no significant quadratic trends were detected (*p* > 0.05; Table 6).

#### 3.4.2. Intestinal Biological Barrier

**Figure 1:**
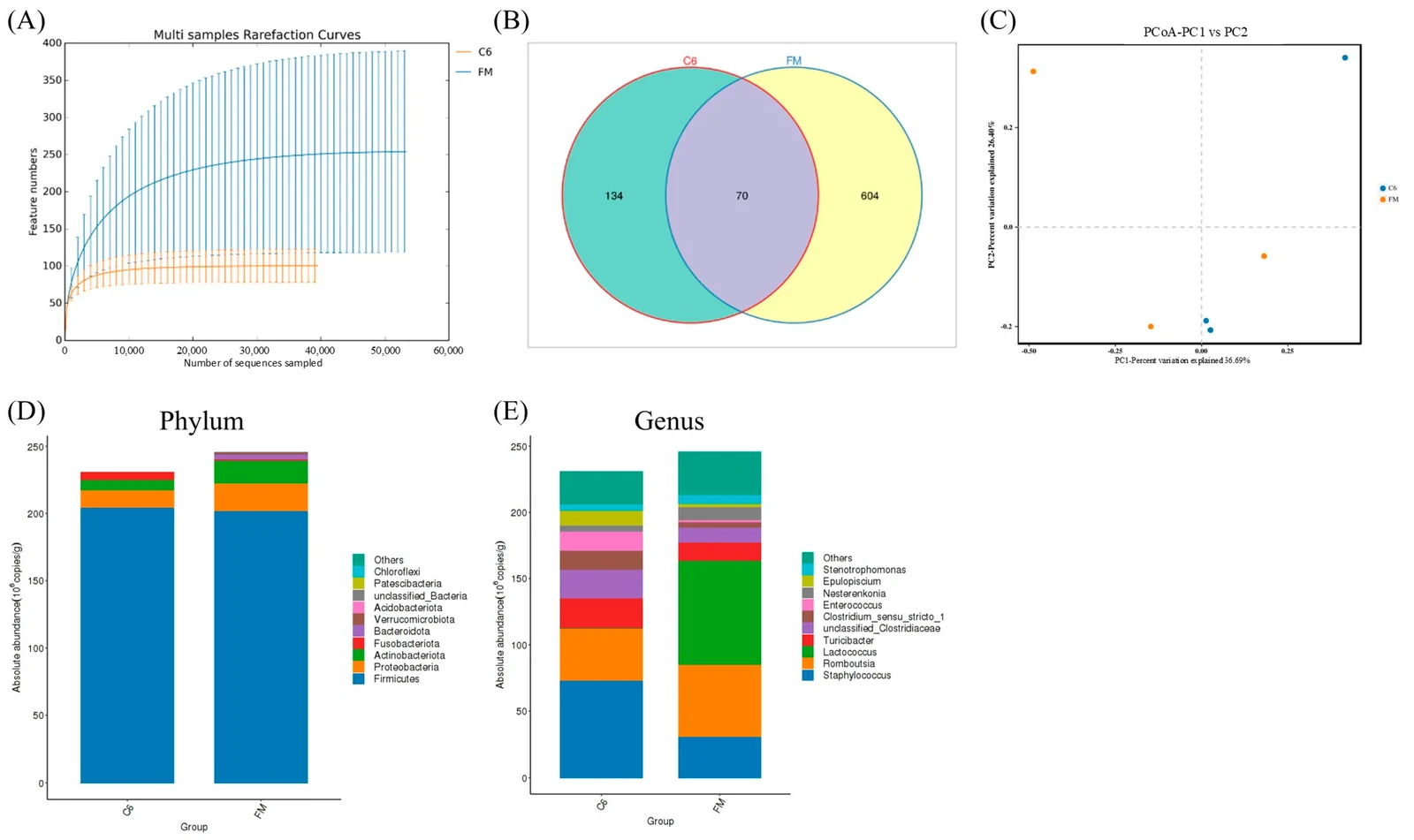
Comparison of hindgut microbial community structure between the FM and C6 groups in hybrid grouper. (**A**) Rarefaction curves of intestinal microbial community; (**B**) Venn diagram showing the distribution of amplicon sequence variants (ASVs); (**C**) principal coordinate analysis (PCoA); (**D**) stacked bar plot of microbial composition at the phylum level; (**E**) stacked bar plot of microbial composition at the genus level.

Hindgut microbiota were analyzed in the FM and C6 groups. A total of 1,112,519 paired-end reads were obtained by 16S rRNA gene sequencing. After quality filtering, paired-end read merging, and sequence filtering, 317,827 high-quality reads were retained. Each sample yielded at least 39,538 high-quality reads, with a mean of 52,971 reads per sample, indicating adequate sequencing depth (Figure 1A). The FM group exhibited higher observed microbial richness than the C6 group. The FM and C6 groups contained 674 and 204 ASVs, respectively, of which 70 were shared between the two groups (Figure 1B). Principal coordinate analysis (PCoA) showed that the first two axes explained 36.69% and 26.40% of the total variation, respectively (Figure 1C).

At the phylum level, the five most abundant taxa were Firmicutes, Proteobacteria, Actinobacteria, Fusobacteria, and Bacteroidota (Figure 1D). Compared with the FM group, the absolute abundances of Proteobacteria and Actinobacteria were lower in the C6 group. At the genus level, the five most abundant genera were *Lactococcus*, *Romboutsia*, *Staphylococcus*, *Turicibacter*, and *Nesterenkonia* (Figure 1E). Compared with the FM group, the absolute abundances of *Staphylococcus* and *Turicibacter* were higher in the C6 group, whereas those of *Romboutsia* and *Lactococcus* were lower.

#### 3.4.3. Intestinal Chemical Barrier

Intestinal MUC2 level did not differ significantly among treatments (FDR *q* > 0.05). Intestinal MPS content and LZM activity progressively decreased with increasing dietary chitin level (FDR *q* < 0.05). However, neither intestinal MPS content nor LZM activity in the C1.5 group differed significantly from that in the FM group. MPS content and LZM activity showed significant negative linear trends. MUC2 also showed a significant negative linear trend despite its nonsignificant overall treatment effect, whereas no significant quadratic trends were detected after FDR correction (Table 7).

#### 3.4.4. Intestinal Physical Barrier

**Figure 2:**
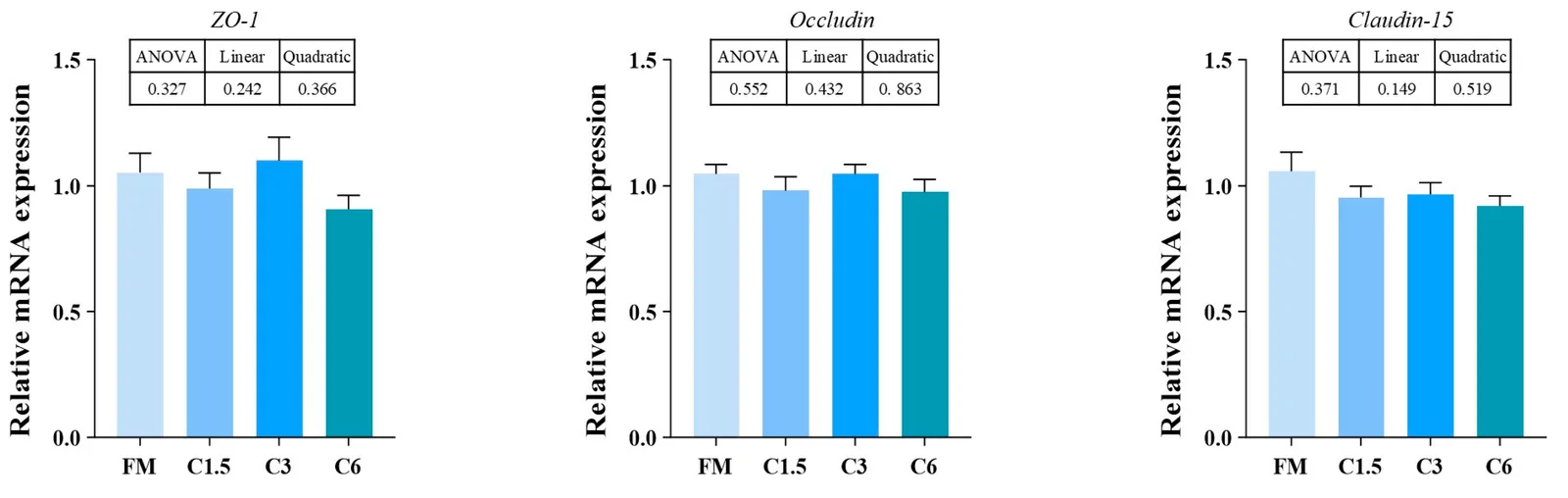
Effects of dietary chitin inclusion on intestinal physical barrier-related indices in hybrid grouper. Values are presented as means ± SEM (*n* = 3). Inset values indicate *p*-values for ANOVA and linear and quadratic trends.

No significant differences were observed in the relative expression levels of intestinal *ZO-1*, *Occludin*, or *Claudin-15* among treatments (*p* > 0.05; Figure 2). Furthermore, none of these genes exhibited significant linear or quadratic dose–response trends with increasing dietary chitin levels (Figure 2).

#### 3.4.5. Intestinal Immune Barrier

Intestinal pIgR content did not differ significantly among treatments (FDR *q* > 0.05). Intestinal IgM, TGF-β1, TNF-α, IFN-γ, IL-1β, IL-6, and IL-8 were significantly reduced by dietary chitin, with the threshold for a significant response varying among indicators (FDR *q* < 0.05). However, intestinal IgM, TGF-β1, IFN-γ, and IL-8 levels in the C1.5 group did not differ significantly from those in the FM group. In contrast, intestinal IL-10 levels progressively increased with dietary chitin inclusion, although neither the C1.5 nor C3 group differed significantly from the FM group. After FDR correction, significant linear trends were detected for IgM, IL-10, TGF-β1, IFN-γ, IL-1β, IL-6, and IL-8, whereas the linear trend for TNF-α was not significant. Only IL-8 exhibited a significant quadratic trend (Table 8).

### 3.5. Serum Biochemical Indices

Serum TG, T-CHO, and ALB levels did not differ significantly among treatments (*p* > 0.05). Compared with the FM group, serum T-BIL content, AST and ALT activities, and the AST/ALT ratio were significantly higher in the C3 and/or C6 groups, depending on the parameter measured. T-BIL, AST, ALT, and the AST/ALT ratio all exhibited significant positive linear trends with increasing dietary chitin levels (*p* < 0.05), whereas no significant quadratic trends were observed (Table 9).

### 3.6. Serum Immune Indices

Serum AKP activity and IgM content did not differ significantly among treatments (FDR *q* > 0.05). Serum LZM activity was significantly increased starting from the C1.5 group, whereas serum complement C3 and C4 levels were significantly elevated only in the C3 and C6 groups. However, serum complement C3 and C4 levels in the C1.5 group did not differ significantly from those in the FM group. After FDR correction, LZM, C3, and C4 exhibited significant positive linear trends (FDR *q* < 0.05), while IgM and LZM showed significant quadratic trends. The quadratic trend in serum IgM was significant despite its nonsignificant overall treatment effect (Table 10).

### 3.7. Hepatic Health

#### 3.7.1. Hepatic Morphological Structure

**Figure 3:**
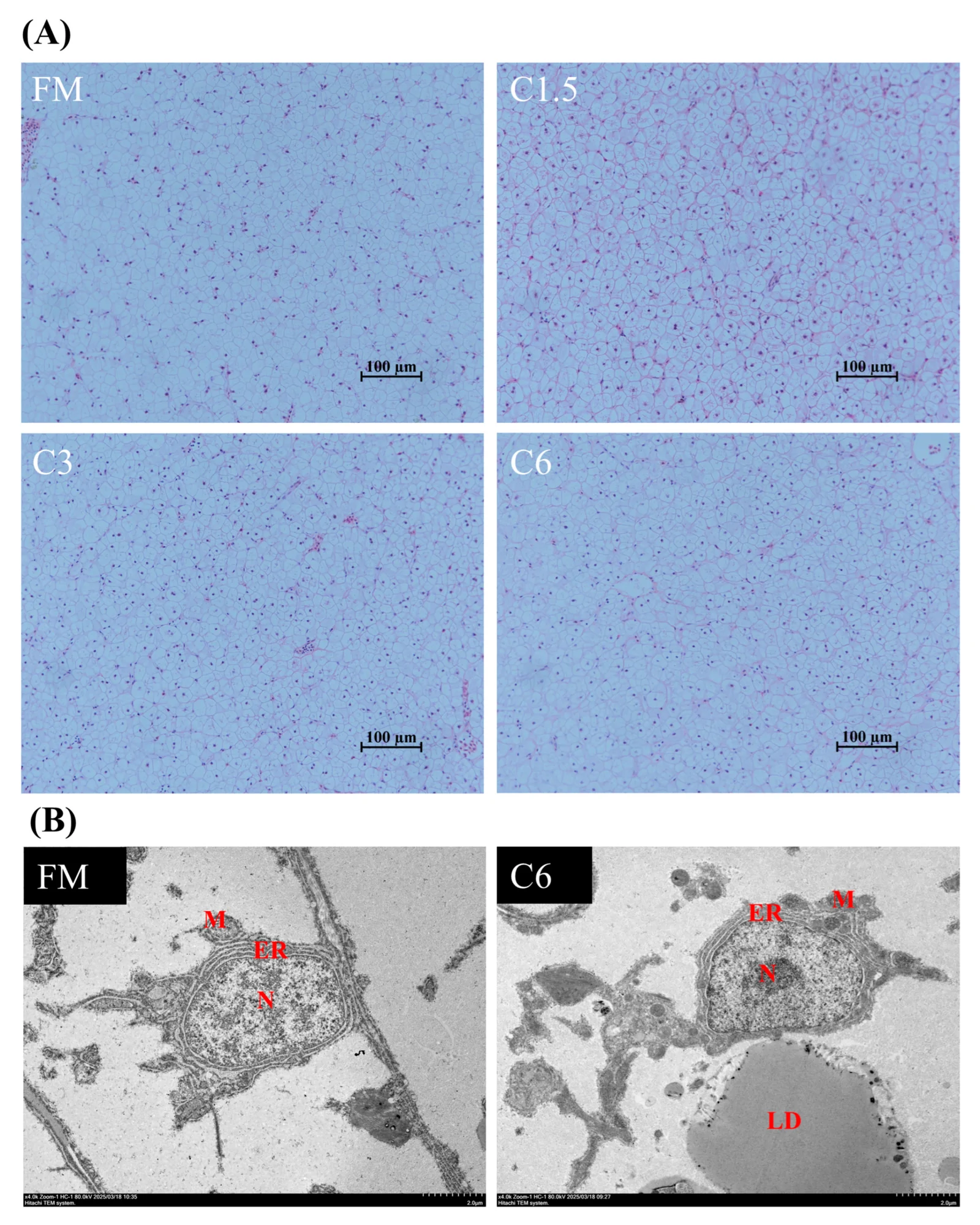
Effects of dietary chitin inclusion on hepatic histology and hepatocyte ultrastructure in hybrid grouper. (**A**) Hematoxylin and eosin (H&E)-stained liver sections from the FM, C1.5, C3, and C6 groups, respectively (100×). (**B**) Transmission electron microscopy (TEM) images of hepatocytes from the FM and C6 groups, respectively (4000×). N, nucleus; ER, endoplasmic reticulum; M, mitochondrion; LD, lipid droplet.

H&E staining showed that the FM group exhibited generally preserved hepatic architecture and relatively regular hepatocyte arrangement, with some hepatocytes displaying cytoplasmic vacuolation. In the C1.5 and C3 groups, hepatocellular vacuolation was also observed, while the overall hepatic architecture remained largely intact. The C6 group showed visible hepatocellular vacuolation and some variation in hepatocyte morphology, although the general tissue organization was maintained (Figure 3A).

Transmission electron microscopy showed that hepatocytes in the FM group exhibited compact endoplasmic reticulum, mitochondria with normal morphology and dense matrices, and regularly arranged cristae. In the C6 group, hepatocytes exhibited slight organelle swelling, irregular cellular morphology, and the presence of lipid droplets (Figure 3B).

#### 3.7.2. Hepatic Immune Function

Hepatic IFN-γ, IL-1β, and IL-8 levels were significantly reduced at 1.5% dietary chitin, whereas LZM, IgM, TGF-β1, TNF-α, and IL-6 levels were significantly reduced at dietary chitin levels of ≥3.0%. In contrast, hepatic IL-10 levels were significantly increased at ≥3.0% dietary chitin, with the highest level observed in the C6 group (FDR *q* < 0.05). After FDR correction, all nine hepatic immune indices exhibited significant linear trends, whereas only IL-8 showed a significant quadratic trend (Table 11).

#### 3.7.3. Hepatic Stress-Regulatory Capacity

**Figure 4:**
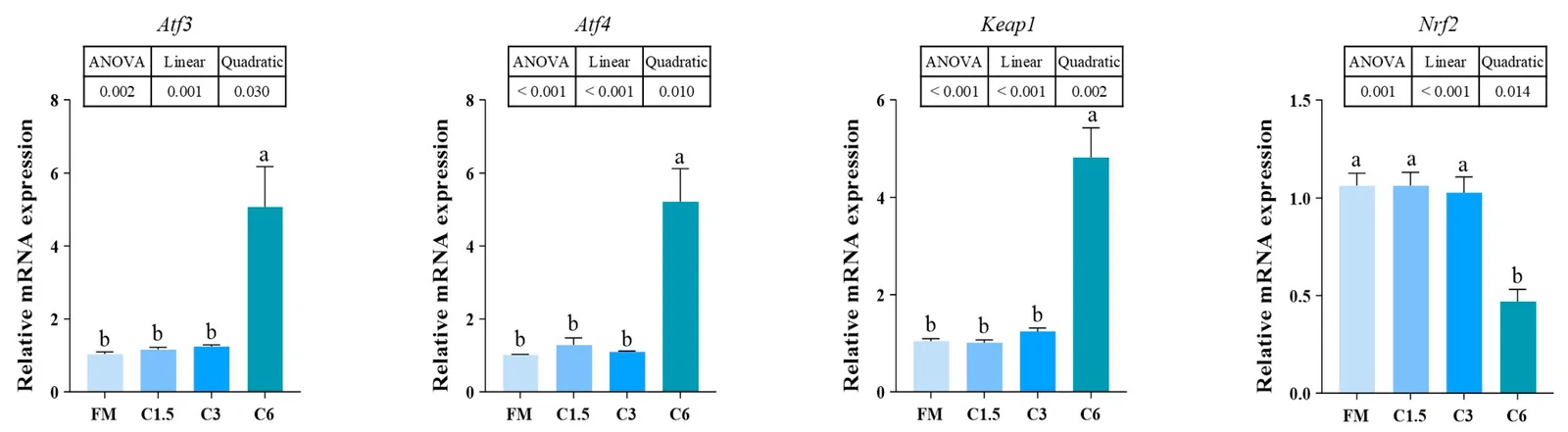
Effects of dietary chitin inclusion on expression of hepatic stress-regulatory genes in hybrid grouper. Values are presented as means ± SEM (*n* = 3). Inset values indicate *p*-values for ANOVA and linear and quadratic trends. Different lowercase letters (a, b) indicate significant differences among treatments (*p* < 0.05).

The relative expression levels of hepatic *Atf3*, *Atf4*, and *Keap1* were significantly higher in the C6 group than in all other groups (*p* < 0.05). In contrast, hepatic *Nrf2* expression was significantly lower in the C6 group than in all other groups. All four genes exhibited significant linear and quadratic dose–response trends with increasing dietary chitin levels (Figure 4).

#### 3.7.4. Hepatic Antioxidant Capacity

Hepatic MDA level was significantly elevated only in the C6 group (*p* < 0.05). In contrast, hepatic T-AOC, CAT activity, and GSH content were significantly lower in the C6 group than in the FM, C1.5, and C3 groups. Hepatic ROS showed a significant overall treatment effect, whereas SOD activity did not differ significantly among treatments (*p* > 0.05). ROS and MDA levels showed significant positive linear trends, whereas T-AOC, CAT, SOD, and GSH showed significant negative linear trends. Significant quadratic trends were also observed for MDA and T-AOC (Table 12).

#### 3.7.5. Hepatocyte Apoptosis

**Figure 5:**
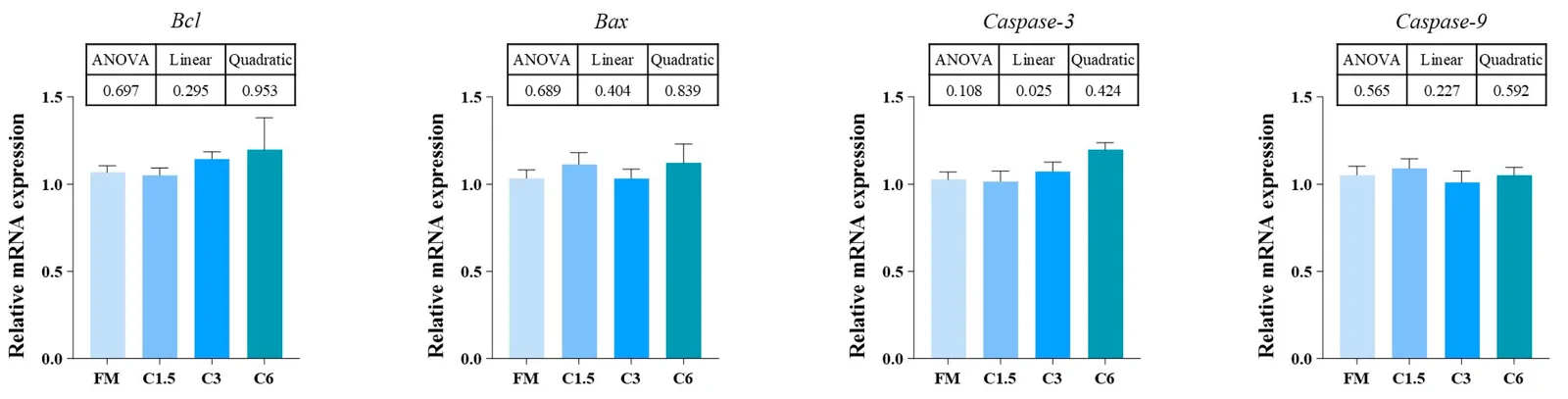
Effects of dietary chitin inclusion on hepatic apoptosis-related indices in hybrid grouper. Values are presented as means ± SEM (*n* = 3). Inset values indicate *p*-values for ANOVA and linear and quadratic trends.

No significant differences were observed in the relative mRNA expression of hepatic *Bcl-2*, *Bax*, *Caspase-3*, or *Caspase-9* among treatments (*p* > 0.05). However, Caspase-3 expression exhibited a significant linear dose–response trend with increasing dietary chitin levels (*p* < 0.05), whereas no significant linear or quadratic trends were detected for the other genes (Figure 5).

### 3.8. Muscle Texture

Muscle hardness, gumminess, and chewiness were significantly reduced by dietary chitin inclusion (*p* < 0.05), whereas adhesiveness, springiness, cohesiveness, and resilience were not significantly affected (*p* > 0.05). Hardness, gumminess, and chewiness showed significant negative linear trends, and hardness and gumminess further exhibited significant quadratic trends (Table 13).

## 4. Discussion

Dietary chitin inclusion at 1.5–6.0% did not significantly affect survival or growth performance but reduced feed utilization efficiency, as indicated by higher FCR and lower PER values. Under the present experimental conditions, dietary chitin therefore appeared to have little effect on somatic growth while reducing feed utilization efficiency in hybrid grouper. Studies in Atlantic halibut (*Hippoglossus hippoglossus*) and Atlantic cod (*Gadus morhua*) have shown that dietary supplementation with up to 5% chitin has only limited effects on growth. In contrast, Atlantic salmon (*Salmo salar*) appear to be more sensitive to dietary chitin, with levels above 1% reportedly impairing growth performance and nutrient utilization [29]. Current evidence indicates that fish responses to chitin-containing ingredients depend strongly on species, ingredient source, and inclusion level. In rainbow trout (*Oncorhynchus mykiss*), dietary chitin levels of 1.5–3.0%, corresponding approximately to 15–30% black soldier fly (*Hermetia illucens*) meal inclusion, were not associated with a significant adverse impact on growth, whereas a chitin level of approximately 4.5%, corresponding to 45% meal inclusion, significantly impaired growth performance. Consistent with these observations, a meta-analysis indicated that dietary inclusion of black soldier fly meal at levels below approximately 29% was generally not associated with impaired growth, whereas the risk of growth depression increased at higher inclusion levels [9,30,31]. Although dietary inclusion of 25% or 50% *Tenebrio molitor* meal, corresponding to approximately 1.2% and 2.3% chitin, respectively, did not impair growth performance in gilthead seabream (*Sparus aurata*), both inclusion levels significantly decreased protein and lipid digestibility [32]. In barramundi (*Lates calcarifer*), dietary inclusion of 46.2% and 61.6% black soldier fly larvae meal resulted in approximately 4.4% and 5.9% dietary chitin, respectively, with higher inclusion negatively affecting growth and feed conversion [33]. In turbot (*Scophthalmus maximus*), inclusion of 16.5% black soldier fly pre-pupae meal, corresponding to approximately 1.47% dietary chitin, was associated with changes in growth performance and chitin-degrading capacity [34]. Collectively, these findings indicate that responses to dietary chitin are strongly species- and context-dependent, with digestive physiology, ingredient matrix, and inclusion level likely contributing to the observed variation. The present study demonstrated altered feed-use performance and changes in selected intestinal enzyme activities in response to dietary chitin, providing a basis for further investigation of its effects on nutrient and chitin digestibility. Nevertheless, no significant differences were observed in whole-body crude protein, crude lipid, ash, or moisture contents among dietary treatments. Notably, the ash content of the experimental diets increased from 13.42% in the FM group to 15.28% in the C6 group with increasing chitin inclusion. Whole-body ash content also increased numerically from 2.38% to 2.72%, although the differences among treatments were not statistically significant. This discrepancy between the dietary and whole-body ash responses may reflect differences in mineral availability, absorption, and retention. However, because the mineral composition of the chitin preparation and the digestibility and retention of dietary minerals were not determined, the relationship between increased dietary ash and whole-body ash content remains unclear.

In fish, the intestine supports nutrient processing and absorption while contributing to mucosal barrier maintenance and immune defense [35]. The intestinal barrier comprises a complex defense system consisting of epithelial cells, tight junctions, the mucus layer, mucosal immunoglobulins, antimicrobial factors, and commensal microbiota. Together, these components regulate nutrient absorption, pathogen exclusion, immune recognition, and microbial homeostasis [36,37,38]. Chitin comprises N-acetylglucosamine residues joined by β-(1 → 4) bonds and contributes to the structural framework of many invertebrates [12]. Its low water solubility, structural stability, and relative resistance to enzymatic hydrolysis may limit its digestibility and utilization in fish. Consequently, physiological responses to dietary chitin may depend partly on species-specific chitinolytic capacity and digestive adaptation. Previous studies have demonstrated marked interspecific variation in chitin utilization. In Atlantic salmon fed diets containing either 6% purified shrimp-shell-derived chitin or approximately 6% chitin supplied by black soldier fly larvae meal, intestinal chitinase activity was not markedly stimulated, suggesting a limited adaptive upregulation of endogenous chitinolytic capacity [16,39]. Nile tilapia (*Oreochromis niloticus*) and rainbow trout fed black soldier fly larvae meal fractions differing in chitin concentration (1.8%, 2.7%, and 15.4% of black soldier fly dry matter) were capable of digesting chitin to some extent; however, apparent chitin digestibility declined as chitin exposure increased [40].

Intestinal Na^+^/K^+^-ATPase and trypsin activities remained unchanged, whereas lipase and chitinase activities declined as dietary chitin level increased. The reduction in hindgut chitinase activity suggests that increased dietary chitin did not induce a compensatory increase in the measured chitinolytic enzyme response. Reduced lipase activity suggests a decline in lipid-digestive capacity. Conversely, the unchanged trypsin and Na^+^/K^+^-ATPase activities indicate that dietary chitin did not uniformly affect intestinal digestive and transport-related functions. Rather, the responses were concentrated in processes associated with chitin degradation and lipid digestion.

These findings are broadly consistent with observations in rainbow trout [29] and partially resemble the intestinal functional disturbances reported in grouper with plant protein-induced enteritis [41,42]. Changes in digesta physicochemical properties and nutrient accessibility have been proposed as possible effects of dietary chitin [15,43]. Although most intestinal and hepatic health-related indices in the C1.5 group remained comparable to those in the FM group, FR and FCR were highest and PER was lowest in this group, with partial recovery at higher inclusion levels, indicating a non-monotonic response. The underlying mechanism remains unclear, as feed palatability, feed refusal, ingestion rate, and nutrient digestibility were not directly evaluated. These findings suggest that dietary chitin inclusion below 1.5% may be more appropriate for maintaining feed utilization efficiency, although further studies are needed to evaluate lower dietary chitin inclusion levels and establish a more appropriate inclusion threshold.

Plasma DAO activity and LPS content were elevated in fish receiving 3% and 6% dietary chitin, consistent with increased intestinal permeability and compromised barrier integrity at higher inclusion levels. Previous studies in fish have used circulating DAO, LPS, and endothelin-related indices as markers associated with intestinal barrier dysfunction and translocation of gut-derived products. In soybean meal-induced enteritis in grouper, these indices similarly increased with increasing intestinal injury [41]. In hybrid grouper, high dietary inclusion of soybean meal, fermented soybean meal, or soy protein concentrate has been associated with intestinal structural alterations, changes in immune-related factors, and shifts in microbial community composition [42,44,45]. These observations suggest that intestinal homeostasis in hybrid grouper may be particularly susceptible to high levels of poorly digestible dietary components or antinutritional factors. Notably, all experimental diets contained cottonseed protein concentrate and soybean meal. Therefore, the observed intestinal responses should be interpreted as changes relative to the common FM control diet.

Notably, dietary chitin did not significantly alter the relative expression of the tight junction-related genes *Occludin*, *ZO-1*, or *Claudin-15*, a response that differs from some observations in plant protein-induced enteritis in hybrid grouper. These findings indicate that changes in intestinal barrier-associated physiological indices were not accompanied by significant transcriptional changes in the tight-junction genes examined [46]. Importantly, unchanged transcription of tight junction-related genes does not necessarily imply preserved barrier function because barrier integrity is regulated at transcriptional, translational, structural, and functional levels.

The intestinal chemical and immune barriers play important roles in maintaining mucosal homeostasis. MUC2 is a major component of the intestinal mucus layer, pIgR participates in mucosal immunoglobulin transport, and LZM contributes to antimicrobial defense [36,47,48]. Lower intestinal MPS and LZM values were observed at higher dietary chitin inclusion levels, whereas MUC2 and pIgR did not differ significantly among treatments. In parallel, several cytokine-related indices were reduced, while IL-10 levels increased in the C6 group. These changes suggest modulation of intestinal immune-related responses and alterations in mucosal immune homeostasis rather than generalized immunosuppression. Importantly, reduced concentrations of individual cytokines do not necessarily indicate impaired immune competence, as such changes may also reflect regulatory adjustments in inflammatory and immune signaling.

The comparison between the FM and C6 groups revealed differences in hindgut microbial richness and community composition at 6.0% dietary chitin inclusion. At the genus level, the C6 group was characterized by reduced absolute abundances of *Lactococcus* and *Romboutsia*, accompanied by increased abundances of *Staphylococcus* and *Turicibacter* relative to the FM group. Lactic acid bacteria such as *Lactococcus* have been associated with nutrient metabolism, mucosal function, and intestinal homeostasis in fish [49,50]. Some *Staphylococcus* species have opportunistic pathogenic potential; therefore, the increased abundance of this genus may indicate altered intestinal ecological conditions, although genus-level taxonomic data alone cannot establish pathogenicity [51,52]. *Turicibacter* has been associated with host immune regulation and nutrient metabolism in several animal models, and its increase in the C6 group may reflect a broader shift in microbial community structure [53,54]. Dietary chitin has also been reported to alter the intestinal microbial community in Atlantic salmon [39]. In fish, the gut microbial community contributes to nutrient acquisition, metabolic balance, immune protection, and resistance to pathogens, while its composition is strongly shaped by dietary inputs. Altered microbial diversity and community composition, together with expansion of potentially unfavorable taxa, are frequently associated with intestinal dysbiosis in fish [55,56]. Accordingly, chitin-associated microbial alterations may have contributed to changes in barrier function and nutrient utilization; however, the present results establish association rather than a causal microbiota-mediated mechanism. Overall, the intestinal responses to dietary chitin were characterized by reduced activities of selected digestive enzymes, weakening of specific chemical- and immune-barrier indices, and differences in microbial community structure between the FM and C6 groups.

The liver is a central organ involved in metabolism, detoxification, and immune regulation in fish and is therefore widely used to assess nutritional and physiological stress responses [57,58]. In the present study, serum T-BIL levels, AST and ALT activities, and the AST/ALT ratio increased significantly at dietary chitin inclusion levels of 3.0% or higher, suggesting that high dietary chitin levels may increase the physiological burden on the liver and induce hepatic stress responses in hybrid grouper. Although serum transaminase levels have been reported in hybrid grouper, validated species-specific reference intervals for these biochemical parameters are currently unavailable. Therefore, elevations in these markers alone are insufficient to confirm hepatocellular injury. Furthermore, changes in hepatic morphology and antioxidant status, particularly those observed at the 6.0% chitin inclusion level, further suggest that high dietary chitin levels may exacerbate hepatic physiological stress [59].

Hepatic H&E staining revealed hepatocyte vacuolation, while transmission electron microscopy showed mild organelle swelling and the presence of lipid droplets in hepatocytes of the C6 group. These morphological findings were consistent with biochemical evidence of hepatic stress at higher dietary chitin levels. However, severe ultrastructural abnormalities, such as marked disruption of mitochondrial cristae or extensive endoplasmic reticulum disorganization, were not evident. These findings indicate that the structural hepatic response to dietary chitin was relatively mild and more consistent with cellular stress than with overt hepatotoxic injury. Few studies have directly examined hepatic histopathology or ultrastructure following purified dietary chitin supplementation in fish. Although chitin-containing insect meals have been associated with alterations in hepatic oxidative status, lipid deposition, and morphology, the specific contribution of chitin cannot be distinguished from that of other nutritional and bioactive components in these ingredients. Consequently, direct comparison with previous studies remains limited. The relatively modest histological alterations observed here therefore suggest that dietary chitin did not induce severe hepatic structural damage under the tested conditions. Although more lipid droplets were observed in the C6 group, no significant differences in whole-body crude lipid content were detected among the dietary treatment groups. This apparent discrepancy may reflect differences between overall whole-body lipid content and localized hepatic lipid deposition. The qualitative TEM observations suggest only a possible alteration in hepatic lipid distribution.

With respect to hepatic antioxidant defense, MDA content increased with dietary chitin inclusion, whereas T-AOC, CAT activity, and GSH content declined at higher inclusion levels. In parallel, hepatic *Atf3*, *Atf4*, and *Keap1* expression increased in the C6 group, whereas *Nrf2* expression decreased, a pattern consistent with enhanced cellular stress signaling and impaired antioxidant regulation. The Keap1-Nrf2 pathway is a major regulator of cellular antioxidant defense, and suppression of Nrf2 signaling is generally associated with reduced antioxidant capacity [60,61]. The upregulation of *Atf3* and *Atf4* further suggests activation of cellular stress-responding signaling, potentially involving pathways associated with endoplasmic reticulum stress. Studies of high-fat diets in fish have shown that hepatic lipid accumulation can activate endoplasmic reticulum stress and disturb lipid metabolism, redox balance, and cellular homeostasis through IRE1-, ATF4-, and ATF6-related pathways [57]. Comparable responses have also been observed in hybrid grouper exposed to dietary MDA or oxidized lipids, where compromised antioxidant capacity was accompanied by greater intestinal permeability and elevated circulating LPS levels [62,63].

In the present study, increased plasma DAO activity and LPS levels, together with impaired hepatic antioxidant status, support a potential association between intestinal barrier disturbance and hepatic stress. Increased intestinal permeability may have increased systemic exposure to gut-derived products, thereby contributing to hepatic oxidative and immune disturbances. Nevertheless, without direct evidence tracing gut-derived factor translocation or subsequent hepatic signaling, a causal mechanism involving the gut–liver axis cannot be confirmed. Hepatic IFN-γ exhibited a progressive decline across the FM, C1.5, C3, and C6 groups, accompanied by increased IL-10 at higher chitin levels. This pattern is consistent with a shift in inflammatory regulation. However, concurrent reductions in hepatic LZM and IgM, together with diminished antioxidant status at 6.0%, suggest broader changes in hepatic immune regulation and cellular stress. Meanwhile, the expression of apoptosis-related genes *Bcl-2*, *Bax*, *Caspase-3*, and *Caspase-9* remained unchanged. Nevertheless, this finding alone cannot determine whether hepatocyte apoptosis was altered. Taken together, the hepatic alterations may have been associated with broader metabolic and immune disturbances accompanying intestinal dysfunction, although the causal sequence linking these responses remains unresolved.

Muscle texture is an important determinant of flesh quality and commercial value in hybrid grouper. Texture parameters provide useful information on muscle mechanical properties and eating quality and are therefore widely used to evaluate market-relevant flesh quality in high-value fish [64,65]. In the present study, increasing dietary chitin reduced muscle hardness, gumminess, and chewiness, whereas adhesiveness, springiness, cohesiveness, and resilience remained unchanged, indicating selective effects on muscle textural properties. Studies directly evaluating the effects of purified dietary chitin on fish muscle texture remain scarce. A recent study in largemouth bass (*Micropterus salmoides*) reported different muscle-quality responses to black soldier fly-derived and shrimp-derived chitin, suggesting that both chitin source and inclusion level may influence physiological outcomes [66]. Although differences in physicochemical properties limit direct extrapolation to Antarctic krill meal, our findings provide useful insights into the physiological responses of hybrid grouper to crustacean-derived chitin and a reference for future krill meal research. Studies using chitin-containing insect meals have likewise reported variable effects on fish muscle quality. Dietary inclusion of defatted *Tenebrio molitor* meal did not significantly alter muscle cellularity or texture in European sea bass (*Dicentrarchus labrax*), whereas high replacement levels of fishmeal with defatted black soldier fly larvae meal affected muscle antioxidant status and water-holding capacity in tongue sole (*Cynoglossus semilaevis*) [67,68]. However, because insect meals also provide proteins, lipids, minerals, and other bioactive compounds, their effects cannot be attributed specifically to chitin. Thus, direct comparisons remain limited; nevertheless, the present results indicate that dietary chitin can alter selected muscle textural properties in hybrid grouper.

Notably, dietary chitin did not alter whole-body proximate composition but reduced muscle hardness, gumminess, and chewiness. This dissociation suggests that the changes in muscle texture cannot be explained solely by alterations in whole-body crude protein or lipid content. Fish muscle texture is influenced by nutrient availability, myofiber development, collagen metabolism, connective tissue organization, and muscle redox status [69,70]. Emerging evidence further suggests that intestinal microbiota and their metabolites may influence fish muscle properties through pathways involving collagen metabolism [71]. Given the concurrent alterations in intestinal digestive function, microbial community structure, and hepatic antioxidant status, the observed changes in muscle texture may be linked to broader disturbances in nutrient utilization and physiological homeostasis. Nevertheless, these potential relationships remain unverified because muscle fiber histology, collagen content, and muscle antioxidant status were not directly examined. Furthermore, cooking loss, water-holding capacity, and sensory characteristics were not evaluated, limiting the assessment of whether the observed reductions in instrumental hardness translate into meaningful changes in processing properties or eating quality. Further studies integrating instrumental texture measurements with muscle structural analysis, physicochemical assessments, and sensory evaluation are needed to clarify the underlying mechanisms and practical significance of these findings.

Although the present study provides a comprehensive assessment of the physiological responses of hybrid grouper to dietary chitin, several limitations should be considered when interpreting the findings. The progressive replacement of wheat starch with chitin, accompanied by changes in dietary ash content, and the physicochemical characteristics of the commercial chitin preparation may have contributed to the observed responses. As nutrient and chitin digestibility and feed palatability were not directly assessed, the mechanisms underlying the non-monotonic changes in feed utilization remain unclear. Furthermore, intestinal responses were evaluated primarily through physiological and biochemical indicators without complementary histological assessment, while microbiota analysis was restricted to the FM and C6 groups. The interpretation of hepatic responses was also limited by the absence of established species-specific biochemical reference ranges and the qualitative nature of morphological observations. In addition, the limited availability of species-specific validation information for certain commercial immunoassays should be considered when interpreting immune-related findings. Instrumental muscle texture measurements alone cannot fully reflect the sensory quality of fish flesh. Future studies incorporating lower dietary chitin inclusion levels and actual Antarctic krill meal are warranted to further clarify appropriate inclusion levels and the practical implications of these findings.

## 5. Conclusions

Dietary chitin inclusion at 1.5% impaired feed utilization and altered muscle textural properties in hybrid grouper, while levels ≥ 3.0% were associated with broader alterations in intestinal and hepatic physiological indices. Although survival and growth performance were not significantly affected, all chitin-containing diets showed poorer feed utilization. Based on our unpublished measurements of Antarctic krill meal chitin (7.90–8.43%), 1.50% dietary chitin theoretically corresponds to 17.8–19.0% krill meal inclusion. These findings provide a reference for krill meal utilization, but do not establish a safe inclusion limit. Further studies using lower chitin levels (<1.5%) and actual krill meal are warranted.

## Figures and Tables

**Table 1:** Formulation and proximate composition of the experimental diets (%, dry-matter basis).

Ingredients	FM	C1.5	C3	C6
Fish meal	50.00	50.00	50.00	50.00
Chitin	0.00	1.53	3.06	6.12
Cottonseed protein concentrate	16.00	16.00	16.00	16.00
Soybean meal	8.00	8.00	8.00	8.00
Wheat flour	7.00	7.00	7.00	7.00
Wheat starch	6.65	5.12	3.59	0.53
Soybean oil	3.50	3.50	3.50	3.50
Fish oil	3.00	3.00	3.00	3.00
Soy lecithin	2.00	2.00	2.00	2.00
Monocalcium phosphate	1.50	1.50	1.50	1.50
Choline chloride (50%)	0.30	0.30	0.30	0.30
Vitamin C	0.03	0.03	0.03	0.03
Vitamin premix ^1^	1.00	1.00	1.00	1.00
Mineral premix ^2^	1.00	1.00	1.00	1.00
Ethoxyquin	0.02	0.02	0.02	0.02
Total	100.00	100.00	100.00	100.00
Proximate composition				
Dry matter (%)	90.31	90.30	91.58	90.82
Crude protein (%)	48.73	48.68	48.69	48.73
Crude lipid (%)	11.23	11.04	11.14	11.22
Ash (%)	13.42	13.63	14.38	15.28
Gross energy (kJ/g)	18.59	18.84	18.37	18.42
Chitin (%)	0.01	1.50	3.03	6.03

Note: ^1^ Vitamin premix composition (per kg of premix): vitamin A, 5000 IU; vitamin D_3_, 1000 IU; vitamin E, 40 mg; thiamine, 5 mg; riboflavin, 10 mg; menadione, 10 mg; pyridoxine, 10 mg; cyanocobalamin, 0.02 mg; niacin, 40 mg; calcium pantothenate, 20 mg; biotin, 0.1 mg; folic acid, 1 mg; vitamin C, 150 mg. The premix was supplied by Guangdong Yuehai Feed Group Co., Ltd. (Zhanjiang, China). ^2^ Mineral premix composition (per kg of premix): iron, 100 mg; copper, 3 mg; zinc, 50 mg; manganese, 12 mg; iodine, 0.8 mg; cobalt, 0.2 mg; selenium, 0.3 mg. The premix was supplied by Guangdong Yuehai Feed Group Co., Ltd. (Zhanjiang, China).

**Table 2:** Primer sequences used for RT-qPCR analysis.

Gene	Primer Sequence (5′−3′)	Accession No.
*ZO-1*	F: TCCACTCCGGTCAAAAGCTC	MK809396.1
	R: GACAGGAAGGTCCACGTCAG	
*Occludin*	F: GGAGGAGAAACAGGGAATGAACT	XM_033622284.1
	R: TCTGCTACAGCCTGGTATTTGG	
*Claudin-15*	F: GGCGGCCCTGATAGGAATAC	XM_033651910.1
	R: GTTGAAGGCGTACCAGGACA	
*Atf3*	F: CCAAACACCCGAGGATGAGAGAAAC	XM_042503803.1
	R: AGGAGGAGGCGGAGGAGGAG	
*Atf4*	F: TGGAGCAGACGATGGCAAAGATG	XM_042485343.1
	R: CGGATGAGCAGGAACCAATGAGG	
*Keap1*	F: TGGAGGCGGTGGACTTTGTG	XM_018665037.1
	R: AGAGATGCTGGCTGTGTAGGC	
*Nrf2*	F: TATGGAGATGGGTCCTTTGGTG	KU892416.1
	R: GCTTCTTTTCCTGCGTCTGTTG	
*Bcl-2*	F: ATCGTAGGGCTTTTCGCTTTC	XM_049582491.1
	R: CTCCCATCCTCTTTGGCTCTG	
*Bax*	F: ACTGGGGAAGAATCATCGTG	XM_033648354.1
	R: CGTCCTGAAGAAATCCAAACA	
*Caspase-3*	F: CGCAAAGAGTAGCGACGGA	XM_049571989.1
	R: CGATGCTGGGGAAATTCAGAC	
*Caspase-9*	F: TTTTCCTGGTTATGTTTCGTGG	XM_049582009.1
	R: TTGCTTGTAGAGCCCTTTTGC	
*β-actin*	F: GGCTACTCCTTCACCACCACA	XM_033645256.1
	R: TCTGGGCAACGGAACCTCT	

Note: *ZO-1*, zonula occludens-1; *Atf3*, activating transcription factor 3; *Atf4*, activating transcription factor 4; *Keap1*, kelch-like ECH-associated protein 1; *Nrf2*, nuclear factor erythroid 2-related factor 2; *Bcl-2*, B-cell lymphoma 2; *Bax*, BCL2-associated X protein; *Caspase-3*, caspase 3; *Caspase-9*, caspase 9.

**Table 3:** Effects of dietary chitin inclusion on growth performance and morphological indices of hybrid grouper.

Items	FM	C1.5	C3	C6	*p*-Value		
					ANOVA	Linear	Quadratic
IBW (g)	15.06 ± 0.02	14.99 ± 0.00	15.03 ± 0.03	15.03 ± 0.03	0.291	0.628	0.223
FBW (g)	111.03 ± 1.40	111.71 ± 3.72	115.74 ± 3.03	112.84 ± 3.48	0.714	0.614	0.431
FR (%/d)	1.32 ± 0.01 ^c^	1.92 ± 0.02 ^a^	1.51 ± 0.01 ^b^	1.49 ± 0.03 ^b^	<0.001	0.796	<0.001
WGR (%)	637.27 ± 8.91	645.08 ± 24.92	670.40 ± 21.42	651.02 ± 24.18	0.719	0.604	0.405
SGR (%/d)	2.38 ± 0.02	2.39 ± 0.04	2.43 ± 0.04	2.40 ± 0.04	0.749	0.629	0.439
FCR	0.74 ± 0.01 ^c^	1.07 ± 0.01 ^a^	0.82 ± 0.00 ^b^	0.83 ± 0.02 ^b^	<0.001	0.587	<0.001
PER	2.64 ± 0.01 ^a^	1.76 ± 0.04 ^c^	2.35 ± 0.04 ^b^	2.31 ± 0.07 ^b^	<0.001	0.251	<0.001
SR (%)	85.56 ± 1.11	82.22 ± 1.11	90.00 ± 5.09	84.44 ± 1.11	0.300	0.894	0.435
HSI (%)	2.77 ± 0.15	2.44 ± 0.11	2.49 ± 0.18	2.64 ± 0.23	0.503	0.813	0.173
VSI (%)	10.86 ± 0.47	11.22 ± 0.32	11.18 ± 0.31	11.94 ± 0.49	0.266	0.107	0.456
ISI (%)	0.77 ± 0.06	0.85 ± 0.05	0.87 ± 0.03	0.85 ± 0.03	0.409	0.312	0.189
CF (g/cm^3^)	2.91 ± 0.08	2.77 ± 0.09	2.75 ± 0.07	3.02 ± 0.06	0.068	0.226	0.018

Data are presented as means ± SEM (*n* = 3). Different superscript letters (a, b and c) within the same row indicate significant differences among treatments (*p* < 0.05). IBW, initial body weight; FBW, final body weight; FR, feeding rate; WGR, weight gain rate; SGR, specific growth rate; FCR, feed conversion ratio; PER, protein efficiency ratio; SR, survival rate; HSI, hepatosomatic index; VSI, viscerosomatic index; ISI, intestinosomatic index; CF, condition factor. Feeding rate (FR, %/d) = dry feed intake/{[(initial body weight + final body weight)/2] × days} × 100; Weight gain rate (WGR, %) = (final body weight − initial body weight)/initial body weight × 100; Specific growth rate (SGR, %/d) = (ln(final body weight) − ln(initial body weight))/days × 100; Feed conversion ratio (FCR) = dry feed intake/(final total body weight − initial total body weight); Protein efficiency ratio (PER) = body weight gain (g)/protein intake (g); Intestinosomatic index (ISI, %) = intestine weight/body weight × 100; Survival rate (SR, %) = final fish number/initial fish number × 100; Hepatosomatic index (HSI, %) = liver weight/body weight × 100; Viscerosomatic index (VSI, %) = visceral weight/body weight × 100; Condition factor (CF, g/cm^3^) =100 × body weight/body length^3^.

**Table 4:** Effects of dietary chitin inclusion on whole-body composition of hybrid grouper (wet-weight basis, %).

Items	FM	C1.5	C3	C6	*p*-Value		
					ANOVA	Linear	Quadratic
Moisture (%)	77.64 ± 0.95	76.96 ± 0.61	76.52 ± 0.73	77.30 ± 0.68	0.607	0.880	0.204
Crude protein (%)	14.48 ± 0.64	14.34 ± 0.19	15.42 ± 0.57	14.43 ± 0.52	0.446	0.883	0.286
Crude lipid (%)	4.50 ± 0.19	5.12 ± 0.07	5.10 ± 0.19	5.03 ± 0.15	0.068	0.091	0.045
Ash (%)	2.38 ± 0.08	2.49 ± 0.03	2.57 ± 0.10	2.72 ± 0.07	0.057	0.010	0.786

Data are presented as means ± SEM (*n* = 3).

**Table 5:** Effects of dietary chitin inclusion on digestive and absorptive enzyme activities in hybrid grouper.

Items	FM	C1.5	C3	C6	*p*-Value		
					ANOVA	Linear	Quadratic
Trypsin (U/mg protein)	170.98 ± 10.60	163.12 ± 6.46	138.71 ± 2.44	143.66 ± 15.58	0.148	0.064	0.254
Lipase (U/g protein)	114.08 ± 4.54 ^a^	118.23 ± 9.86 ^a^	107.28 ± 6.28 ^a^	82.09 ± 2.32 ^b^	0.016	0.004	0.161
Chitinase (U/g protein)	9.49 ± 0.59 ^a^	6.69 ± 0.13 ^b^	7.22 ± 0.69 ^b^	6.00 ± 0.40 ^b^	0.006	0.003	0.092
Na^+^/K^+^-ATPase (U/mg protein)	128.10 ± 19.35	159.13 ± 18.04	184.59 ± 4.07	188.32 ± 7.32	0.051	0.015	0.149

Data are presented as means ± SEM (*n* = 3). Different superscript letters (a, b) within the same row indicate significant differences among treatments (*p* < 0.05). Trypsin, lipase, and Na^+^/K^+^-ATPase activities were measured in the foregut, whereas chitinase activity was measured in the hindgut.

**Table 6:** Effects of dietary chitin inclusion on plasma indices related to intestinal mucosal permeability in hybrid grouper.

Items	FM	C1.5	C3	C6	*p*-Value		
					ANOVA	Linear	Quadratic
LPS (EU/L)	387.66 ± 9.20 ^c^	437.60 ± 47.67 ^bc^	491.86 ± 32.65 ^b^	598.22 ± 15.19 ^a^	0.006	0.001	0.960
ET-1 (pg/mL)	75.69 ± 3.89	83.77 ± 4.02	87.84 ± 6.18	91.00 ± 5.41	0.230	0.065	0.416
DAO (U/mL)	16.32 ± 0.71 ^b^	16.62 ± 0.42 ^b^	22.21 ± 0.91 ^a^	23.93 ± 1.93 ^a^	0.003	0.001	0.490

Data are presented as means ± SEM (*n* = 3). Different superscript letters (a, b and c) within the same row indicate significant differences among treatments (*p* < 0.05). LPS, lipopolysaccharide; ET-1, endothelin-1; DAO, diamine oxidase. All parameters were measured in heparinized plasma.

**Table 7:** Effects of dietary chitin inclusion on intestinal chemical barrier indices in hybrid grouper.

Items	FM	C1.5	C3	C6	FDR *q*-Value		
					ANOVA	Linear	Quadratic
MPS (mg/g protein)	215.89 ± 12.01 ^a^	208.48 ± 1.70 ^a^	175.82 ± 6.32 ^b^	105.38 ± 10.68 ^c^	<0.001	<0.001	0.145
MUC2 (pg/mg protein)	156.53 ± 12.93	137.03 ± 17.11	133.43 ± 10.89	99.70 ± 8.09	0.071	0.026	0.892
LZM (U/g protein)	86.96 ± 2.34 ^a^	83.17 ± 2.56 ^a^	73.97 ± 1.71 ^b^	57.46 ± 2.31 ^c^	<0.001	<0.001	0.407

Data are presented as means ± SEM (*n* = 3). Different superscript letters (a, b and c) within the same row indicate significant differences among treatments after FDR correction (FDR *q* < 0.05). MPS, mucopolysaccharide; MUC2, mucin 2; LZM, lysozyme.

**Table 8:** Effects of dietary chitin inclusion on intestinal immune barrier indices in hybrid grouper.

Items	FM	C1.5	C3	C6	FDR *q*-Value		
					ANOVA	Linear	Quadratic
IgM (μg/mg protein)	442.23 ± 8.40 ^a^	430.27 ± 3.75 ^a^	358.97 ± 4.72 ^b^	308.41 ± 3.37 ^c^	<0.001	<0.001	0.546
pIgR (μg/mg protein)	64.61 ± 3.29	53.16 ± 9.00	47.21 ± 1.40	46.47 ± 3.09	0.114	0.074	0.223
IL-10 (pg/mg protein)	90.55 ± 3.95 ^b^	95.00 ± 14.50 ^b^	123.05 ± 16.56 ^ab^	148.61 ± 11.75 ^a^	0.048	0.030	0.940
TGF-β1 (pg/mg protein)	57.41 ± 4.68 ^a^	50.20 ± 5.33 ^ab^	41.72 ± 0.48 ^b^	38.44 ± 4.07 ^b^	0.048	0.032	0.309
TNF-α (pg/mg protein)	222.01 ± 9.20 ^a^	164.05 ± 10.82 ^b^	177.12 ± 8.50 ^b^	167.77 ± 16.66 ^b^	0.042	0.065	0.095
IFN-γ (pg/mg protein)	25.88 ± 1.65 ^a^	21.82 ± 0.34 ^a^	17.36 ± 1.94 ^b^	15.00 ± 0.48 ^b^	0.007	0.003	0.140
IL-1β (pg/mg protein)	32.23 ± 1.64 ^a^	24.19 ± 3.09 ^b^	25.59 ± 1.53 ^b^	21.57 ± 1.16 ^b^	0.042	0.032	0.283
IL-6 (pg/mg protein)	58.39 ± 2.67 ^a^	48.02 ± 3.03 ^b^	42.45 ± 1.74 ^b^	39.54 ± 3.43 ^b^	0.019	0.009	0.095
IL-8 (pg/mg protein)	41.24 ± 2.46 ^a^	35.82 ± 0.85 ^ab^	31.67 ± 2.18 ^b^	33.77 ± 0.59 ^b^	0.042	0.046	0.049

Data are presented as means ± SEM (*n* = 3). Different superscript letters (a, b and c) within the same row indicate significant differences among treatments after FDR correction (FDR *q* < 0.05). IgM, immunoglobulin M; pIgR, polymeric immunoglobulin receptor; IL-10, interleukin-10; TGF-β1, transforming growth factor-β1; TNF-α, tumor necrosis factor-α; IFN-γ, interferon-γ; IL-1β, interleukin-1β; IL-6, interleukin-6; IL-8, interleukin-8.

**Table 9:** Effects of dietary chitin inclusion on serum biochemical indices in hybrid grouper.

Items	FM	C1.5	C3	C6	*p*-Value		
					ANOVA	Linear	Quadratic
TG (mmol/L)	4.11 ± 0.94	3.81 ± 0.68	4.25 ± 0.38	2.85 ± 0.16	0.427	0.198	0.441
T-CHO (mmol/L)	8.70 ± 1.77	6.94 ± 1.75	6.84 ± 1.14	8.90 ± 1.67	0.708	0.792	0.283
ALB (g/L)	5.36 ± 0.42	5.14 ± 0.54	6.17 ± 0.14	6.01 ± 0.42	0.287	0.166	0.660
T-BIL (μmol/L)	11.33 ± 0.61 ^b^	11.27 ± 0.58 ^b^	13.60 ± 0.26 ^a^	14.83 ± 0.30 ^a^	0.001	<0.001	0.947
AST (U/L)	56.02 ± 1.50 ^c^	57.69 ± 2.72 ^c^	109.32 ± 4.55 ^b^	124.76 ± 4.15 ^a^	<0.001	<0.001	0.070
ALT (U/L)	34.99 ± 1.40 ^b^	35.56 ± 3.40 ^b^	46.93 ± 1.86 ^a^	43.45 ± 1.65 ^a^	0.012	0.011	0.078
AST/ALT	1.60 ± 0.05 ^c^	1.64 ± 0.13 ^c^	2.34 ± 0.16 ^b^	2.88 ± 0.16 ^a^	<0.001	<0.001	0.845

Data are presented as means ± SEM (*n* = 3). Different superscript letters (a, b and c) within the same row indicate significant differences among treatments (*p* < 0.05). TG, triglyceride; T-CHO, total cholesterol; ALB, albumin; T-BIL, total bilirubin; AST, aspartate aminotransferase; ALT, alanine aminotransferase.

**Table 10:** Effects of dietary chitin inclusion on serum immune indices in hybrid grouper.

Items	FM	C1.5	C3	C6	FDR *q*-Value		
					ANOVA	Linear	Quadratic
AKP (KU/dL)	8.54 ± 0.81	8.17 ± 1.21	8.60 ± 0.77	10.15 ± 0.80	0.474	0.266	0.499
IgM (mg/mL)	2.08 ± 0.12	1.81 ± 0.08	1.70 ± 0.06	1.89 ± 0.01	0.058	0.267	0.028
LZM (U/L)	216.71 ± 20.12 ^c^	370.76 ± 5.06 ^b^	365.07 ± 12.91 ^b^	484.43 ± 1.74 ^a^	<0.001	<0.001	0.033
C3 (μg/mL)	77.77 ± 10.78 ^b^	76.41 ± 7.83 ^b^	103.59 ± 8.44 ^a^	129.31 ± 0.47 ^a^	0.007	0.002	0.582
C4 (μg/mL)	131.21 ± 5.11 ^c^	135.67 ± 4.30 ^c^	220.99 ± 6.09 ^b^	240.96 ± 7.72 ^a^	<0.001	<0.001	0.056

Data are presented as means ± SEM (*n* = 3). Different superscript letters (a, b and c) within the same row indicate significant differences among treatments after FDR correction (FDR *q* < 0.05). AKP, alkaline phosphatase; IgM, Immunoglobulin M; LZM, lysozyme; C3, complement 3; C4, complement 4.

**Table 11:** Effects of dietary chitin inclusion on hepatic immune indices in hybrid grouper.

Items	FM	C1.5	C3	C6	FDR *q*-Value		
					ANOVA	Linear	Quadratic
LZM (U/g protein)	76.12 ± 3.41 ^a^	70.80 ± 4.85 ^a^	54.53 ± 1.52 ^b^	50.65 ± 3.18 ^b^	0.003	0.001	0.235
IgM (μg/mg protein)	392.30 ± 20.47 ^a^	394.47 ± 2.55 ^a^	263.81 ± 5.09 ^b^	269.37 ± 22.50 ^b^	0.001	<0.001	0.109
IL-10 (pg/mg protein)	59.99 ± 3.83 ^c^	59.80 ± 0.86 ^c^	74.27 ± 2.57 ^b^	92.55 ± 3.83 ^a^	0.001	<0.001	0.302
TGF-β1 (pg/mg protein)	39.14 ± 3.00 ^a^	36.35 ± 0.60 ^a^	27.51 ± 0.77 ^b^	23.42 ± 2.02 ^b^	0.002	0.001	0.338
TNF-α (pg/mg protein)	134.62 ± 7.86 ^a^	120.60 ± 5.31 ^ab^	103.85 ± 2.51 ^bc^	91.71 ± 6.93 ^c^	0.005	0.002	0.328
IFN-γ (pg/mg protein)	14.86 ± 0.64 ^a^	13.01 ± 0.03 ^b^	10.56 ± 0.73 ^c^	8.28 ± 0.55 ^d^	0.001	<0.001	0.302
IL-1β (pg/mg protein)	19.59 ± 1.99 ^a^	15.40 ± 0.39 ^b^	14.74 ± 0.55 ^b^	10.51 ± 1.19 ^c^	0.005	0.002	0.553
IL-6 (pg/mg protein)	31.34 ± 1.35 ^a^	30.29 ± 0.79 ^a^	24.81 ± 1.19 ^b^	20.90 ± 1.12 ^c^	0.001	<0.001	0.764
IL-8 (pg/mg protein)	26.72 ± 1.42 ^a^	22.54 ± 0.47 ^b^	15.37 ± 0.11 ^c^	15.26 ± 1.00 ^c^	<0.001	<0.001	0.004

Data are presented as means ± SEM (*n* = 3). Different superscript letters (a, b, c and d) within the same row indicate significant differences among treatments after FDR correction (FDR *q* < 0.05). LZM, lysozyme; IgM, immunoglobulin M; IL-10, interleukin-10; TGF-β1, transforming growth factor-β1; TNF-α, tumor necrosis factor-α; IFN-γ, interferon-γ; IL-1β, interleukin-1β; IL-6, interleukin-6; IL-8, interleukin-8.

**Table 12:** Effects of dietary chitin inclusion on hepatic antioxidant indices in hybrid grouper.

Items	FM	C1.5	C3	C6	*p*-Value		
					ANOVA	Linear	Quadratic
ROS (RFU/mg protein)	419.17 ± 28.55 ^b^	455.40 ± 41.82 ^b^	457.60 ± 35.84 ^b^	577.47 ± 18.62 ^a^	0.039	0.008	0.423
MDA (nmol/mg protein)	4.76 ± 0.82 ^b^	4.98 ± 0.57 ^b^	5.97 ± 0.27 ^b^	10.49 ± 0.51 ^a^	<0.001	<0.001	0.035
T-AOC (μmol/mg protein)	27.53 ± 0.49 ^a^	26.84 ± 1.33 ^a^	26.06 ± 1.56 ^a^	15.13 ± 1.21 ^b^	<0.001	<0.001	0.014
CAT (U/mg protein)	47.14 ± 1.50 ^a^	46.72 ± 1.11 ^a^	47.08 ± 1.48 ^a^	38.12 ± 2.43 ^b^	0.014	0.004	0.076
SOD (U/mg protein)	146.02 ± 4.64	145.61 ± 1.67	147.14 ± 1.81	136.69 ± 1.84	0.095	0.038	0.157
GSH (μg/mg protein)	111.87 ± 3.60 ^a^	112.15 ± 3.56 ^a^	108.72 ± 2.18 ^a^	95.21 ± 2.01 ^b^	0.011	0.002	0.143

Data are presented as means ± SEM (*n* = 3). Different superscript letters (a, b) within the same row indicate significant differences among treatments (*p* < 0.05). ROS, reactive oxygen species; MDA, malondialdehyde; T-AOC, total antioxidant capacity; CAT, catalase; SOD, superoxide dismutase; GSH, glutathione.

**Table 13:** Effects of dietary chitin inclusion on muscle texture properties in hybrid grouper.

Items	FM	C1.5	C3	C6	*p*-Value		
					ANOVA	Linear	Quadratic
Hardness (g)	876.89 ± 51.15 ^a^	635.56 ± 74.09 ^b^	530.79 ± 44.49 ^bc^	444.72 ± 7.30 ^c^	0.002	<0.001	0.036
Adhesiveness (g·s)	−7.12 ± 2.42	−3.86 ± 0.85	−2.97 ± 0.19	−4.39 ± 2.25	0.403	0.376	0.162
Springiness (mm)	0.27 ± 0.03	0.26 ± 0.02	0.30 ± 0.02	0.23 ± 0.03	0.399	0.368	0.331
Cohesiveness	0.19 ± 0.01	0.17 ± 0.02	0.19 ± 0.01	0.18 ± 0.03	0.677	0.937	0.568
Gumminess (g)	169.12 ± 3.04 ^a^	105.20 ± 14.81 ^b^	98.78 ± 6.49 ^b^	81.46 ± 10.05 ^b^	0.001	<0.001	0.013
Chewiness (g·mm)	46.42 ± 4.48 ^a^	26.98 ± 3.42 ^b^	29.77 ± 4.00 ^b^	19.77 ± 4.79 ^b^	0.012	0.004	0.175
Resilience	0.11 ± 0.01	0.09 ± 0.01	0.10 ± 0.01	0.09 ± 0.01	0.155	0.110	0.468

Data are presented as means ± SEM (*n* = 3). Different superscript letters (a, b and c) within the same row indicate significant differences among treatments (*p* < 0.05).

## Data Availability

The raw 16S rRNA gene sequencing data generated in this study have been deposited in the NCBI Sequence Read Archive (SRA) under BioProject accession number PRJNA1549773. Other data supporting the findings of this study are available from the corresponding author upon reasonable request.
